# Improving the pH-stability of Versatile Peroxidase by Comparative Structural Analysis with a Naturally-Stable Manganese Peroxidase

**DOI:** 10.1371/journal.pone.0140984

**Published:** 2015-10-23

**Authors:** Verónica Sáez-Jiménez, Elena Fernández-Fueyo, Francisco Javier Medrano, Antonio Romero, Angel T. Martínez, Francisco J. Ruiz-Dueñas

**Affiliations:** 1 Centro de Investigaciones Biológicas, CSIC, Madrid, Spain; 2 Department of Biotechnology, Delft University of Technology, Delft, The Netherlands; Russian Academy of Sciences, Institute for Biological Instrumentation, RUSSIAN FEDERATION

## Abstract

Versatile peroxidase (VP) from the white-rot fungus *Pleurotus eryngii* is a high redox potential peroxidase of biotechnological interest able to oxidize a wide range of recalcitrant substrates including lignin, phenolic and non-phenolic aromatic compounds and dyes. However, the relatively low stability towards pH of this and other fungal peroxidases is a drawback for their industrial application. A strategy based on the comparative analysis of the crystal structures of VP and the highly pH-stable manganese peroxidase (MnP4) from *Pleurotus ostreatus* was followed to improve the VP pH stability. Several interactions, including hydrogen bonds and salt bridges, and charged residues exposed to the solvent were identified as putatively contributing to the pH stability of MnP4. The eight amino acid residues responsible for these interactions and seven surface basic residues were introduced into VP by directed mutagenesis. Furthermore, two cysteines were also included to explore the effect of an extra disulfide bond stabilizing the distal Ca^2+^ region. Three of the four designed variants were crystallized and new interactions were confirmed, being correlated with the observed improvement in pH stability. The extra hydrogen bonds and salt bridges stabilized the heme pocket at acidic and neutral pH as revealed by UV-visible spectroscopy. They led to a VP variant that retained a significant percentage of the initial activity at both pH 3.5 (61% after 24 h) and pH 7 (55% after 120 h) compared with the native enzyme, which was almost completely inactivated. The introduction of extra solvent-exposed basic residues and an additional disulfide bond into the above variant further improved the stability at acidic pH (85% residual activity at pH 3.5 after 24 h when introduced separately, and 64% at pH 3 when introduced together). The analysis of the results provides a rational explanation to the pH stability improvement achieved.

## Introduction

Lignin degradation has been a hot topic of research for several decades, and still actual nowadays. It is a key step for carbon recycling in land ecosystems and also a central issue for the industrial utilization of lignocellulosic biomass as a renewable feedstock [1]. In nature, the so-called white-rot fungi belonging to the group of basidiomycetes are unique due to their ability to degrade lignin from plant biomass in an efficient way. This process begins with the unspecific oxidative attack to the aromatic units of this polymer by means of a battery of extracellular oxidoreductases among which ligninolytic peroxidases play a key role [2].

Manganese peroxidase (MnP, EC 1.11.1.13) and lignin peroxidase (LiP, EC 1.11.1.14) are two families of ligninolytic heme peroxidases described 30-years ago [3, 4]. The first one is characterized by having a Mn-binding site, formed by three acidic residues (two glutamates and one aspartate) and the internal propionate of heme, where Mn^2+^ is oxidized [5]. The resulting Mn^3+^ acts as a diffusible oxidizer after being chelated by organic acids secreted by white-rot fungi. This metal cation can directly oxidize the (minor) phenolic substructures of lignin and indirectly generate lipid peroxyl radicals able to oxidize the non-phenolic units of this polymer [6]. Two MnP subfamilies have been identified. Long/extralong MnPs are specific for Mn^2+^ [7], whereas members of the short MnP subfamily are also able to oxidize phenols like generic peroxidases (GP, EC 1.11.1.7) [8]. Unlike MnP, LiP displays a catalytic tryptophan exposed to the solvent involved in direct oxidation of a such bulky and heterogeneous substrate as lignin is [9, 10]. Versatile peroxidase (VP, EC 1.11.1.16) constitutes the third family of ligninolytic peroxidases, which was described 20-years ago [11, 12]. VP combines catalytic properties of the above two families due to the presence of both a Mn-oxidation site [13] and a catalytic tryptophan [14] in its molecular structure. This peroxidase also exhibits characteristics of GPs by its ability to oxidize low redox potential substrates (e.g. phenols) at the main heme access channel [15].

As a consequence of their wide substrate specificity, ligninolytic peroxidases are able to oxidize not only lignin but also other phenolic and non-phenolic aromatic compounds and different industrial dyes, revealing that these enzymes have a high industrial interest [1, 16]. They are suitable and attractive for different applications such as the production of biofuels, materials and chemicals of added value in lignocellulosic biorefineries, for the bleaching process in the paper pulp manufacture and for the treatment of dye wastewater [1, 17, 18]. However, their high biotechnological potential cannot be exploited because some difficulties prevent their industrial application. Some of these drawbacks are insufficient levels of protein production and instability towards different factors such as pH, temperature or hydrogen peroxide concentration [1, 19].

In recent years, several genomes of basidiomycete species involved in plant biomass biodegradation have been sequenced [20] and the number is increasing. As a result, the sequences of different types of peroxidases have been identified in these genomes and subsequently expressed and characterized. Some of them have new structural, catalytic and stability properties [7, 8, 21, 22]. Among these, MnP4 from the *Pleurotus ostreatus* genome exhibits a remarkably high acidic and alkaline stability [8]. The study of this and other new peroxidases will provide us with valuable information about the relationships existing among the structure, the stability and the catalytic properties of these enzymes that will allow the design of new biocatalysts of interest.

In the present work, VP (isoenzyme VPL2) from *Pleurotus eryngii* has been subjected to protein engineering using a rational design strategy. The crystal structures of *P*. *eryngii* VP and *P*. *ostreatus* MnP (isoenzyme MnP4 following the genome nomenclature) were compared, and putative stabilizing motifs responsible for the high stability towards pH of this MnP were identified. Subsequently, these motifs and other generally accepted stabilizing structural determinant (i.e. one disulfide bond) were translated to VP with the aim of increasing its pH stability and obtaining a more adequate biocatalyst for industrial applications. The results here presented demonstrate that the use of structural determinants identified in peroxidases obtained from genomic analysis is a useful tool for designing biocatalysts of interest.

## Materials and Methods

### Chemicals

Isopropyl-β-D-thiogalactopyranoside (IPTG), dithiothreitol (DTT), hemin, oxidized glutathione (GSSG), veratryl alcohol (VA), manganese(II) sulphate, Reactive Black 5 (RB5), 2,6-dimethoxyphenol (DMP), sodium tartrate and other chemicals were purchased from Sigma-Aldrich; urea and hydrogen peroxide were from Merck; and 2,2'-azino-bis(3-ethylbenzothiazoline-6-sulfonate) (ABTS) from Roche.

### Design of VP Variants

VPi and VPi-br variants were designed *in silico* based on a comparative analysis of the mature *P*. *eryngii* VP (allelic variant VPL2; GenBank^™^ AF007222) and *P*. *ostreatus* MnP4 (ID 1099081 in the *P*. *ostreatus* PC15 v2.0 genome sequence from the Joint Genome Institute, JGI, at http://genome.jgi.doe.gov/PleosPC15_2/PleosPC15_2.home.html). For this analysis: i) the amino acid sequence alignment of both enzymes was performed using the pairwise sequence alignment tools (Needle, Stretcher, Water and Matcher programs) available at the European Bioinformatics Institute (EMBL-EBI); and ii) the structural alignment of VPL2 (PDB: 2BOQ) and MnP4 (PDB: 4BM1) was carried out with PyMOL (http://pymol.org). From this analysis, the VPi coding sequence was prepared by replacing codons encoding eight amino acid residues in VPL2 with those present at homologous positions in MnP4. The substituted amino acids were Asp69 → Ser (TCC), Thr70 → Asp (GAC), Ser86 → Glu (GAG), Asp146 → Thr (ACC), Gln202 → Leu (CTC), His232 → Glu (GAG), Ser301 → Lys (AAG) and Gln239 → Arg (CGC). The introduction of the following additional mutations in VPi resulted in the VPi-br variant: Thr2 → Lys (AAG), Ala131 → Lys (AAG), Gln219 → Lys (AAA), Leu288 → Arg (CGT), Ala308 → Arg (CGC), Ala309 → Lys (AAG) and Ala314 → Arg (CGT). Both VPi and VPi-br sequences were synthesized by ATG:biosynthetics (Merzhausen, Germany) and cloned into the *Nde*I/*Bam*HI restriction sites of the expression vector pFLAG1 (International Biotechnologies Inc., Cambridge, UK).

Other two VP variants were produced using the QuikChange^™^ Site-Directed Mutagenesis kit (Stratagene, La Jolla, CA, USA). Each of them was obtained by mutagenic PCR using the expression vector pFLAG1 containing the VPi (pFLAG1-VPi) or the VPi-br (pFLAG1-VPi-br) coding sequences as template, and two primers consisting of a direct and a reverse oligonucleotide designed complementary to opposite strands of the same DNA region containing the desired mutations. To obtain VPi-ss (A49C/A61C), a first PCR was carried out using pFLAG1-VPi as template and primers for A49C mutation, 5’-CC CTT CGT TTG ACT TTC CAC GAT TGC ATC GGT TTC TCT CC-3' (only direct sequences are shown here and below, with the changed triplets underlined and the mutations introduced in bold). Then, a second PCR was made using pFLAG1-VPi containing the A49C mutation as template and primers for A61C mutation, 5’-GGC GGA GGA GGA TGT GAC GGT TCC ATC ATC GCG-3’. VPi-br-ss was obtained using pFLAG1-VPi-br as template and primers for A49C and A61C mutations in two consecutive PCR reactions.

PCR reactions were carried out in an Eppendorf Mastercycler Pro S using 10 ng of template DNA, 250 μM each dNTP, 125 ng of both direct and reverse primers, 2.5 units of *Pfu* Turbo DNA polymerase AD (Stratagene) and the manufacture´s reaction buffer. Reaction conditions were as follows: (i) a “hot start” of 95°C for 1 min; (ii) 18 cycles at 95°C for 50 s, 55°C for 50 s and 68°C for 10 min; and (iii) a final cycle at 68°C for 10 min. The plasmids obtained from the mutagenic PCR were transformed into *Escherichia coli* DH5α for plasmid propagation. At least one positive clone of each variant was completely sequenced using an ABI 3730 DNA Analyzer (Applied Biosystem), checked and used to transform *E*. *coli* W3110 for protein expression.

### Enzyme Production, Activation and Purification

Native (i.e. wild-type recombinant enzyme) VP of *P*. *eryngii* (isoenzyme VPL2) and its mutated variants were expressed in *E*. *coli* W3110 after transformation with the corresponding expression vectors. Cells were grown in Terrific Broth medium [23] supplemented with 100 μg/ml of ampicillin at 37°C and 180 rpm until OD_500_~1 (~3 h). Then protein expression was induced with 1 mM IPTG and cells were grown for a further 4 h. The apoenzyme accumulated in inclusion bodies and was recovered by solubilization in 50 mM Tris-HCl (pH 8.0) containing 8 M urea, 1 mM EDTA and 1 mM DTT for 1 h at 4°C. The subsequent *in vitro* folding of the solubilized protein was performed overnight at room temperature in a solution of 0.16 M urea, 20 μM hemin, 5 mM CaCl_2_, 0.5 mM GSSG, 0.1 mM DTT and 0.1 mg/ml protein in 20 mM Tris-HCl at pH 9.5. The refolded enzyme was purified by Resource-Q chromatography using a 0–0.3 M NaCl gradient (2 ml/min flow rate, 20 min) in 10 mM sodium tartrate (pH 5.5) containing 1 mM CaCl_2_. Finally the enzymes were dialyzed against 10 mM sodium tartrate (pH 5). The purified native VP and its variants showed an *R*_*z*_ value (A_407_/A_280_)~ 4 indicative of their high purity. Moreover, the UV-visible spectrum obtained in the 300–700 nm range was used to check the correct incorporation of heme into the enzyme [24]. Enzyme concentration was determined from the absorbance of the Soret band (ε_407_ = 150 mM^-1^ cm^-1^) [12].

### Kinetic Studies

Oxidation of Mn^2+^ was estimated by the formation of Mn^3+^-tartrate complex (ε_328_ = 6500 M^-1^ cm^-1^) in 0.1 M sodium tartrate (pH 5) and that of VA by formation of veratraldehyde (ε_310_ = 9300 M^-1^ cm^-1^) in the same buffer at pH 3 and 2.5. Oxidation of ABTS was followed by generation of its cation radical (ε_436_ = 29300 M^-1^ cm^-1^) and that of RB5 by its disappearance (ε_598_ = 30000 M^-1^ cm^-1^) both in 0.1 M tartrate buffer at pH 3.5 (RB5 also at pH 3). H_2_O_2_ concentration was determined using ε_240_ = 39.4 M^-1^ cm^-1^ [25]. All enzymatic activities were measured as initial velocities taking linear increments (decreases for RB5) in the presence of 0.1 mM H_2_O_2_ using a Shimadzu UV-1800 spectrophotometer. Values and standard errors for apparent affinity constant (Michaelis constant, *K*_m_) and maximal enzyme turnover (catalytic constant, *k*_cat_) were obtained fitting the experimental measurements to the Michaelis-Menten model with SigmaPlot 12.0 software (Systat. Software Inc, California). Fitting of these constants to the normalized Michaelis-Menten equation *υ* = (*k*_*cat*_/*K*_m_)[S]/(1+[S]/*K*_m_) yielded enzyme efficiency values (*k*_*cat*_/*K*_m_) with their standard errors.

### Optimum pH Determination

The optimum pH for oxidation of different substrates was determined by measuring the enzymatic activity of the native VP and its mutated variants at saturating concentrations of VA (20 mM), RB5 (15 μM) and ABTS (7 mM) in 0.1 M Britton-Robinson (B&R) buffer [26], and Mn^2+^ (6 mM) in 0.1 M sodium tartrate over the pH range 2.5–5.5, using 0.1 mM H_2_O_2_ and 0.01–0.03 μM enzyme.

### pH Stability Studies

The enzymes were incubated at pH 3, 3.5 and 7 in 0.1 M B&R buffer at 25°C for 120 h. Their residual activity was estimated after 1 min, 1 h, 4 h, 24 h and 120 h incubation by measuring the oxidation of ABTS (2 mM) using 0.1 mM H_2_O_2_ and 0.01 μM of enzyme in 0.1 M sodium tartrate (pH 3.5). The activity obtained with the sample incubated for 1 min at pH 5 was taken as reference (maximum activity). UV-visible spectra in the 300–700 nm range and absorbance at the Soret band (407 nm) were recorded during the incubation at pH 3, 3.5 and 7 by using an Agilent 8453 diode array spectrophotometer.

### Thermal Stability Studies

The enzymes were incubated at different temperatures (30–80°C) in 10 mM sodium tartrate (pH 5) for 10 min using a Thermo Shaker TS-100 BIOSAN, and then chilled on ice for 5 min. Their residual activity was measured by ABTS (2 mM) oxidation using 0.1 mM H_2_O_2_ and 0.01μM enzyme in 0.1 M sodium tartrate (pH 3.5), at 25°C. The activity of the enzyme measured before incubation at each temperature was taken as reference (maximum activity) to calculate the percentage of residual activity. Data of residual activity for each temperature were fitted to a sigmoidal model and T_50_ values (temperature at which the activity is half of the initial one after 10 minutes of incubation) were calculated from the fit.

### Crystallization

Crystallization trials were carried out by the sitting drop vapor diffusion method, using 96-well MRC2 plates with 50 μl reservoir solution and the commercially available screenings from Emerald (Wizard classic crystallization screens I, II and III) and Jena Biosciences (JBScreen Classic Kits 1–10). Drops consisted of 0.2 μl of protein solution (10 mg/ml in 10 mM sodium tartrate buffer at pH 5.0) and 0.2 μl of reservoir solution. Crystallization was carried out at 22°C. Crystals suitable for X-ray data collection were obtained from these initial trials. Crystals of the mutant VPi were obtained in 0.1 M sodium MES buffer at pH 6.5, 25% PEG 4000 and 0.2 M MgCl_2_; and cryoprotected with 20% glycerol. Crystals of the mutant VPi-br were obtained in 10% PEG 4000 and 20% 2-Propanol; and cryoprotected with Paratone-N oil. Crystals of the mutant VPi-ss were obtained in 0.1 M sodium citrate buffer at pH 5.4, 12% PEG 4000 and 0.2 M calcium acetate; and cryoprotected with Paratone-N oil.

### Data Collection and Processing

Crystals were mounted in nylon loops and flash-frozen in liquid nitrogen in the mother liquor containing the cryoprotectant indicated above. All diffraction data were obtained at 100 K. X-ray diffraction intensities were collected at SOLEIL (Gyf-sur-Yvette, France) and ALBA (Barcelona, Spain) synchrotrons. Diffraction data were indexed, integrated, merged and scaled using the program XDS [27]. Data collection statistics are shown in Table 1.

**Table 1:** Data collection and refinement statistics.

Data collection	VPi	VPi-br	VPi-ss
Space group	P 2_1_ 2_1_ 2_1_	P 2_1_ 2_1_ 2_1_	P 2_1_ 2_1_ 2_1_
Cell constants	a = 46.6, b = 68.5, c = 84.9	a = 54.8, b = 64.2, c = 95.4	a = 45.9, b = 68.8, c = 87.8
Resolution range (Å)	50.00–2.20 (2.33–2.20)	50.00–1.10 (1.16–1.10)	50.00–2.30 (2.43–2.30)
N° of total reflections	183536	1331552	134131
N° of unique reflections	14447	135701	12985
R_merge_ (%)	9.1 (76.9)	6.9 (109.4)	12.0 (56.5)
Completeness (%)	99.9 (99.3)	97.9 (86.7)	99.9 (99.5)
<I/σ(I)>	18.6 (3.2)	15.3 (1.2)	13.9 (3.2)
Multiplicity	12.7 (13.0)	9.8 (6.0)	10.3 (10.7)
CC(1/2)	99.9 (97.4)	99.9 (56.0)	99.8 (97.2)
Solvent content (%) / Matthews coef.	35.27 / 1.90	47.96 / 2.36	40.87 / 2.08
Subunits per asymmetric unit	1	1	1
Wilson B factor (Å^2^)	46.9	15.7	46.3
Refinement			
Resolution range	50.0–2.20 Å	50.0–1.10 Å	50.00–2.30 Å
Working reflections	14361	135667	12942
R_work_ / R_free_	20.6 / 27.5%	13.4 / 14.4%	24.8 / 30.0%
Protein atoms (non H)	2407	2360	2315
Heme group	1	1	1
Ca^2+^	2	2	4
Water molecules	31	449	65
Mg^2+^ ions	2	-	-
Mean B factors (Å^2^)			
Protein atoms (non H)	67.70	15.69	64.24
Heme group	36.66	11.78	36.64
Ca^2+^	48.03	10.44	57.35
Water molecules	47.71	27.32	48.27
Mg^2+^ ions	51.11	-	-
Deviations from ideality			
rmsd bond lengths	0.010 Å	0.010 Å	0.003 Å
rmsd angles	1.337°	1.454°	0.772°
Ramachandran plot statistics			
Preferred %	91.67	97.76	91.94
Allowed %	5.25	2.24	7.74
Outliers %	3.09	0.00	0.32
PDB code	5ABN	5ABO	5ABQ

The structures of the three mutants were solved by molecular replacement using the crystal structure of *P*. *eryngii* VPL (3FMU) as the search model and the program PHASER implemented in the PHENIX package [28].

The final models were obtained by consecutive rounds of refinement, performed with the PHENIX package; followed by manual model building, performed with Coot [29] using σ_A_ weighted 2Fo-Fc and Fo-Fc electron density maps. Solvent molecules were introduced in the structure automatically in the refinement as implemented in the PHENIX package and visually inspected. A total of 5% of reflections was used to calculate the R_free_ value throughout the refinement process. The structures were validated using MolProbity [30, 31]. Refinement and final model statistics are summarized in Table 1. The coordinates and structure factors have been deposited with the Protein Data Bank accession codes 5ABN, 5ABO and 5ABQ. All figures were produced with PyMOL.

## Results

### Rational Design Strategy

**Fig 1:**
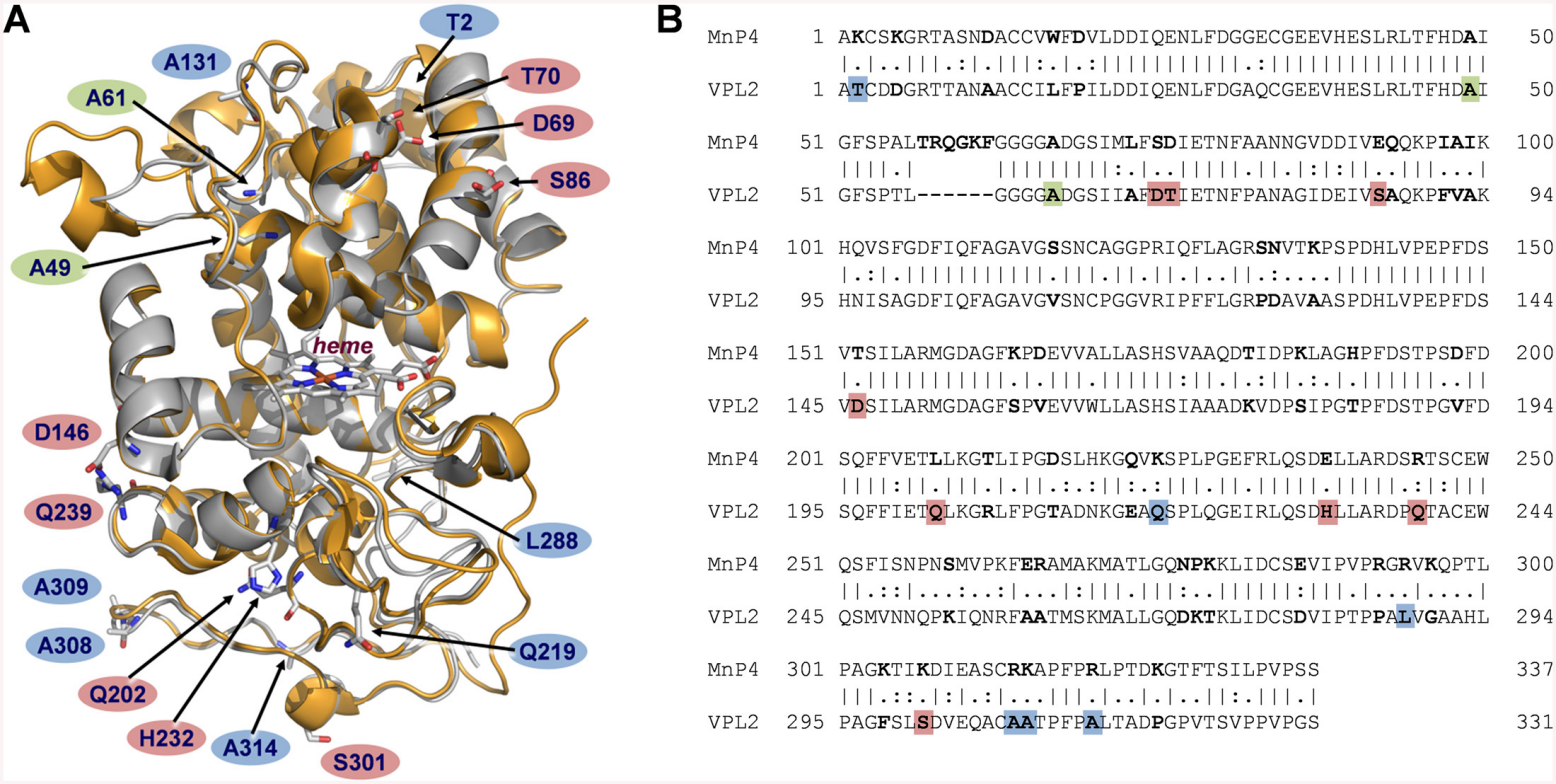
Structural and amino acid sequence alignment of VP (isoenzyme VPL2) from *P*. *eryngii* and MnP4 from *P*. *ostreatus*. (**A**) Superimposition of VP (PDB 2BOQ) (grey) and MnP4 (PDB 4BM1) (orange) crystal structures (shown as cartoons) highlighting the VP amino acid residues mutated in this work (shown together with the heme group as CPK-colored sticks, and labeled according to the color code described below); and (**B**) alignment of their amino acid sequences (labeled using the same color code) (vertical lines denote conserved residues, and colons and periods indicate conservative and semi-conservative substitutions, respectively). Residues explored in the structural comparative analysis of VP and MnP4 searching for putative stabilizing motifs are shown in bold in the amino acid sequence alignment. VP amino acids subsequently substituted with those of MnP4 to generate the VPi variant appear on red background; those substituted by basic residues present in MnP4, introduced into VPi to form the VPi-br variant, are shown on blue background; and alanines substituted by cysteines to form an extra disulfide bridge in VPi resulting in the VPi-ss variant are highlighted on green background.

*P*. *eryngii* VP (isoenzyme VPL2) and *P*. *ostreatus* MnP4 share a common structural scaffold (Fig 1A). Their crystal structures (PDB entries 2BOQ for VP, and 4BM1 for MnP4) superimpose with a root mean square deviation (rmsd) of only 0.75 Å between the C_α_ positions over 316 amino acid residues, covering 95% of the mature proteins. This high structural similarity between both proteins was the basis of our strategy aimed to improve the pH stability of VP, which consisted in identifying the stabilizing motifs putatively contributing to the high stability towards pH of MnP4, and their subsequent transfer into VP. First, the amino acid sequences of these two enzymes were aligned (a 63% sequence identity was found) (Fig 1B). They differ in 124 amino acids, 27 being charged residues in MnP4 and non-charged in VP (while VP only has 11 charged residues being neutral in MnP4). In order to identify those contributing to pH stability in MnP4, a comparative analysis of their position in the molecular structures and interaction with surrounding residues was performed by walking on the superimposed crystal structures of MnP4 and VP on the protein surface. The internal regions of the structures were not explored and subsequent changes at these locations were not performed in VP, with only one exception as described below. Our idea was to substitute only a few amino acid residues in the VP molecular structure minimizing the impact on the catalytic sites to maintain the activity. It is well known that a very subtle equilibrium between stability and activity exits and the improvement of one of these properties is often at the expense of the other.

**Fig 2:**
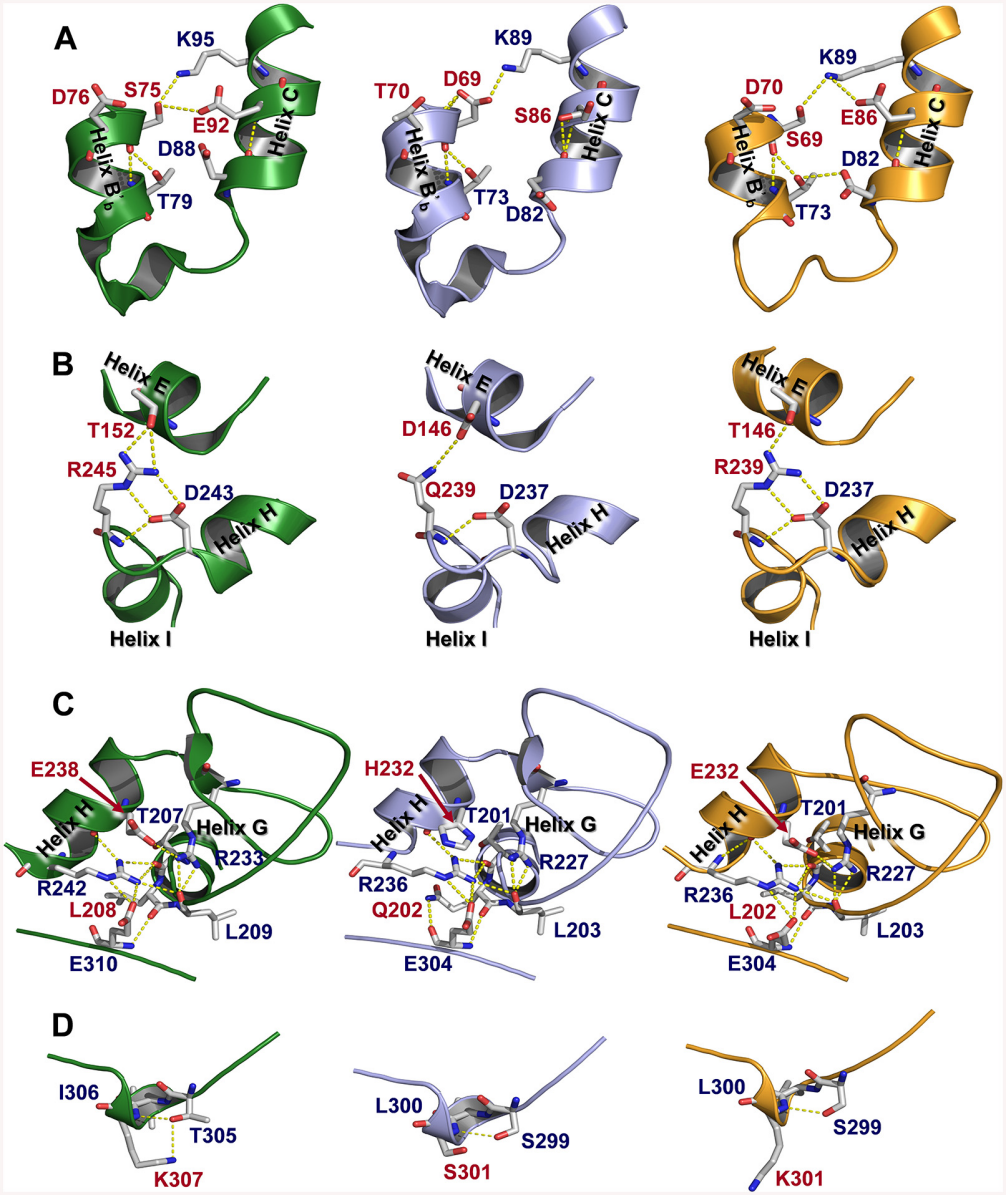
Structural details of four solvent exposed regions (A, B, C and D) in MnP4 (left column), VP (middle column) and VPi variant (right column). Residues mutated in VPi and their homologous in MnP4 and VP are highlighted in red color.

Four regions exposed to the solvent were identified in the MnP4 molecular structure (Fig 2, left column) as hotspots for rational design of a VP with improved stability. These regions exhibit extra ion pairs and hydrogen bond networks in MnP4, compared with VPL2 (Fig 2, middle column), which are responsible for strengthening helix-helix, loop-helix and intra-loop interactions. Based on these observations and considering that most ion pairs have a stabilizing role [32], a VP variant (VPi) containing eight substitutions (D69S/T70D/S86E/D146T/Q202L/H232E/Q239R/S301K) was engineered by introducing the residues involved in these interactions in the four targeted regions. After verifying its increased pH stability (studies described below), new putative stabilizing residues were searched in MnP4. A high number of basic residues with their side chains exposed to the solvent, most of them with no movement restrictions by interactions with surrounding amino acids, were identified in MnP4 (31 of a total of 34 present in the protein, including 20 lysines and 11 arginines). Seven of these exposed residues (4 lysines and 3 arginines) were introduced into VPi, and the VPi-br variant containing the 8 mutations present in VPi plus mutations T2K/A131K/Q219K/L288R/A308R/A309R/A314R was obtained.

A third approach to improve the stability of VP was the further structural stabilization of the distal Ca^2+^-binding site, responsible for maintaining the relative position of the distal histidine involved in enzyme activation by H_2_O_2_. For that, the VPi-ss variant was designed by adding a double mutation (A49C/A61C) to VPi. The two cysteines added to this variant should form an extra disulfide bond contributing to the structural stabilization of the loop containing two of the four amino acid residues that coordinate the distal Ca^2+^ ion. Finally, the VPi-br-ss variant was designed by combining all the mutations described above in a single VP molecule.

The four purified VP variants exhibited the characteristic UV-visible absorption spectrum of the native VP showing relative maxima at 407 nm (Soret band), and at 505 and 637 nm (charge transfer bands CT2 and CT1, respectively) (S1 Fig), which is indicative of an active peroxidase with a high-spin ferric heme [14]. These results proved the correct heme incorporation in the recombinant enzymes.

### Effect of the Mutations on VP Catalytic Properties

**Fig 3:**
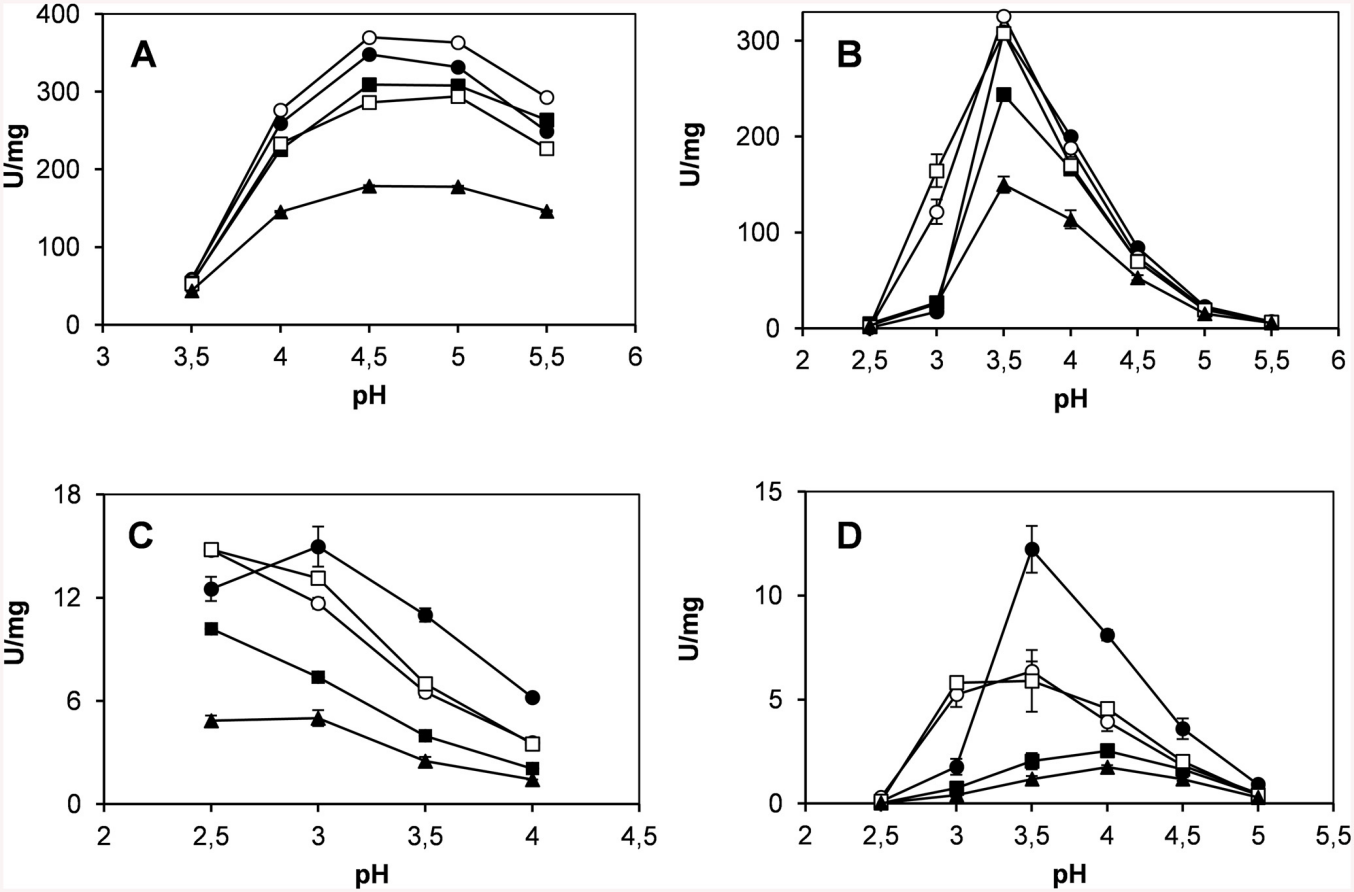
Optimum pH for oxidation of Mn^2+^ (A), ABTS (B), VA (C) and RB5 (D) by native VP (●), VPi (○), VPi-br (■), VPi-ss (□) and VPi-br-ss (▲). Reactions in 0.1 M B&R buffer for ABTS (7 mM), VA (20 mM) and RB5 (15 mM) oxidation; and 0.1 M sodium sodium tartrate for Mn^2+^ oxidation (pH 2.5–5.5), with 0.1 mM H_2_O_2_ at 25°C. Means and 95% confidence limits are shown.

Native VP and the designed variants were kinetically characterized at the three catalytic sites characteristic of this ligninolytic peroxidase (Mn^2+^ oxidation site, main heme access channel and catalytic Trp exposed to the solvent) [16] (Table 2). The optimum pH for oxidation of four different substrates was also determined (Fig 3), in both cases with the aim of identifying potential effects on the catalytic activity due to the mutations introduced. Three of the four variants exhibited a catalytic efficiency for Mn^2+^ oxidation similar to that of the native enzyme. VPi-br-ss was the most affected variant, with only a 40% decrease in efficiency, and all of them (including native VP) showed the same pH activity profile with the optimum at pH 4.5–5 (Fig 3A). With respect to the catalytic activity at the main heme access channel, the optimum pH (3.5) for ABTS oxidation did not experience any variation in the four variants (Fig 3B), although their catalytic efficiency suffered a 35–70% decrease at this pH. The activity of native VP (and that of VPi-br and VPi-br-ss) oxidizing ABTS was dramatically reduced at pH 3 (Fig 3B). By contrast, VPi and VPi-ss showed high activity levels with this substrate at this pH, and a 2.7 and 2.3-fold improved catalytic efficiency, respectively, compared with the native enzyme at its optimum pH (Table 2). Finally, the catalytic activity at the exposed Trp164 responsible for the oxidation of high redox potential substrates was characterized using VA (simple lignin model compound) and RB5 (recalcitrant diazo dye) as reducing substrates. VPi, VPi-ss and VPi-br shifted their optimum pH from 3 to 2.5 for VA oxidation (Fig 3C), and VPi and VPi-ss widened the optimum pH range with RB5 (between pH 3 and 3.5) (Fig 3D). In addition, with both substrates, the catalytic efficiency of VPi and VPi-ss at the lower pH values (pH 2.5 and 3 for VA and RB5 oxidation, respectively) was higher than that of the native enzyme at its optimum pH (Table 2). This effect was more significant for RB5 oxidation, mainly due to a ~8-fold increased affinity (*K*_m_ = 0.4 μM for these variants *vs* 3.4 μM for the native enzyme), and much less important for VA oxidation (*k*_cat_ /*K*_m_ increasing from 2.2 s^-1^ mM^-1^ to 3.4 and 3.6 s^-1^ mM^-1^ in VPi and VPi-ss, respectively).

**Table 2:** Steady-state kinetic constants [*k*_cat_ (s^-1^), *K*_m_ (μM), *k*_cat_ /*K*_m_(s^-1^ mM^-1^)] of native VP and mutated variants for oxidation of Mn^2+^ (at the Mn-binding site), ABTS (at the main heme access channel), and VA and RB5 (at the catalytic Trp exposed to the solvent)^a^.

		VP	VPi		VPi-br	VPi-ss		Vpi-br-ss
**Mn** ^**2+**^	***k***_**cat**_	211 ± 4	217 ± 3		160 ± 2	170 ± 2		133 ± 2
	***K***_**m**_	130 ± 11	106 ± 6		120 ± 7	83 ± 5		134 ± 1
	***k***_**cat**_ **/*K***_**m**_	**1641 ±132**	**2055 ± 106**		**1340 ± 64**	**2041 ± 112**		**997 ± 73**
**ABTS**	***k***_**cat**_	208 ± 6	103 ± 4	73 ± 2 ^b^	125 ± 4	216 ± 15	264 ± 9 ^b^	104 ± 5
	***K***_**m**_	1020 ± 74	797 ± 86	131 ± 12 ^b^	1800± 150	2710 ± 380	575 ± 49 ^b^	1660 ± 180
	***k***_**cat**_ **/*K***_**m**_	**204 ± 10**	**130 ± 10**	**558 ± 39** ^**b**^	**69 ± 4**	**80 ± 6**	**459 ± 27** ^**b**^	**62 ± 4**
**VA**	***k***_**cat**_	5.8 ± 0.1	7.5 ± 0.2	11.1 ± 0.3 ^b^	4.7 ± 0.1	9.8 ± 0.2	8.9 ± 0.3 ^b^	3.1 ± 0.2
	***K***_**m**_	2600 ± 190	4000 ± 390	3250 ± 300 ^b^	5350 ± 470	3960 ± 250	2470 ± 270 ^b^	3850 ± 800
	***k***_**cat**_ **/*K***_**m**_	**2.2 ± 0.1**	**1.9 ± 0.1**	**3.4 ± 0.2** ^**b**^	**0.9 ± 0.1**	**2.5 ± 0.1**	**3.6 ± 0.3** ^**b**^	**0.8 ± 0.1**
**RB5**	***k***_**cat**_	5.5 ± 0.3	4.5 ± 0.1	8.4 ± 0.3 ^b^	3.3 ± 0.2	5.5 ± 0.3	9.0 ± 0.2 ^b^	3.5 ± 0.2
	***K***_**m**_	3.4 ± 0.3	1.4 ± 0.1	0.4 ± 0.05 ^b^	2.1 ± 0.3	3.2 ± 0.4	0.45 ± 0.03 ^b^	1.8 ± 0.2
	***k***_**cat**_ **/*K***_**m**_	**1.6 ± 0.1**	**3.1 ± 0.1**	**19.3 ± 1.8** ^**b**^	**1.5 ± 0.1**	**1.7 ± 0.1**	**19.9 ± 1.0** ^**b**^	**2.0 ± 0.2**

^*a*^ Reactions at 25°C in 0.1 M sodium tartrate (pH 5 for Mn^2+^, pH 3.5 for ABTS and RB5, and pH 3 for VA). Means and 95% confidence limits are shown.

^*b*^ Kinetic constants of VPi and VPi-ss were also measured at pH 3 for oxidation of ABTS and RB5, and at pH 2.5 for oxidation of VA.

### pH and Thermal Stability of VP Variants

**Fig 4:**
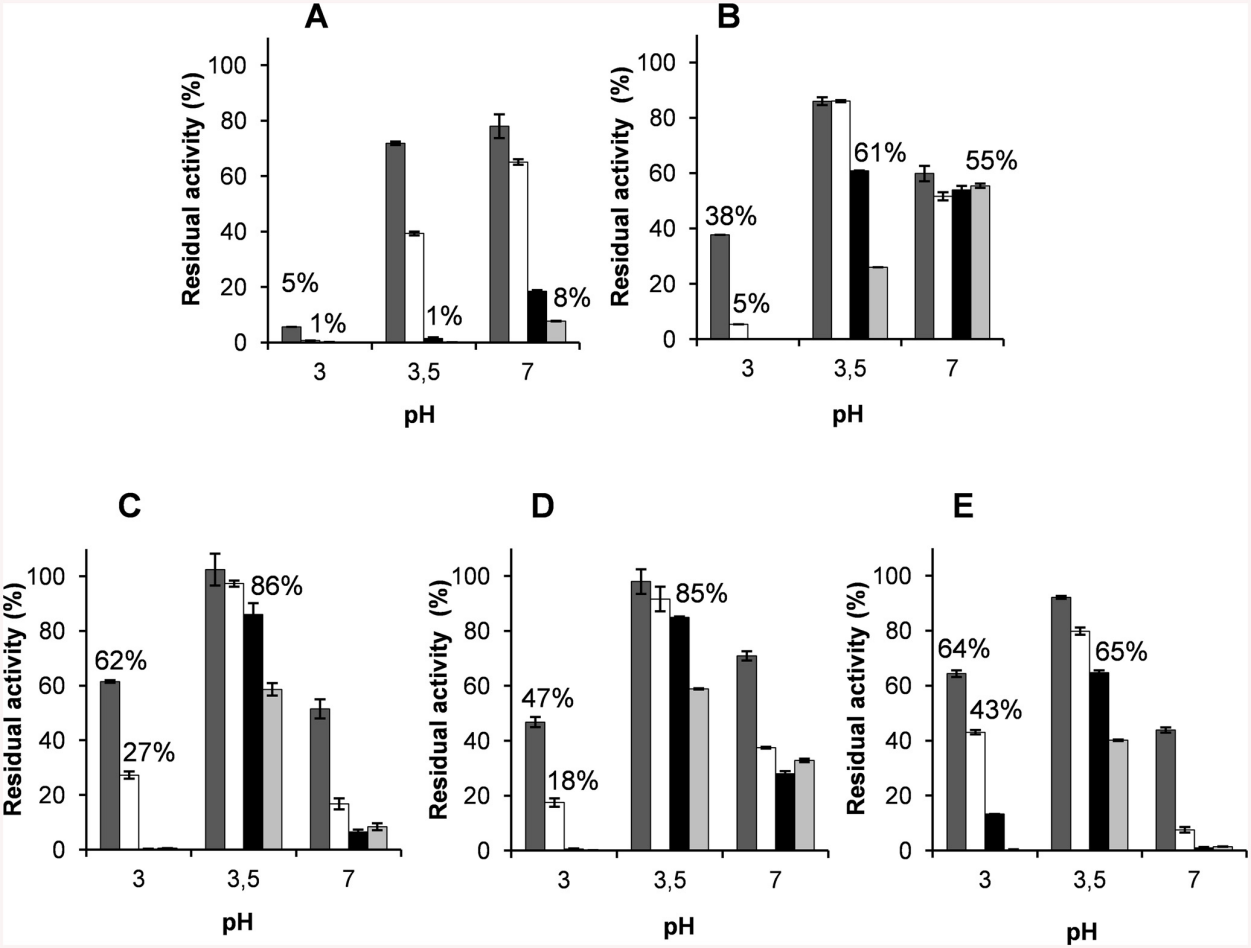
pH stability of native VP (A) and mutated variants VPi (B), VPi-br (C), VPi-ss (D) and VPi-br-ss (E). The enzymes were incubated in 0.1 M B&R buffer (at pH 3, 3.5 and 7) at 25°C, and their residual activity was measured after 1 (∎), 4 (□), 24 (■) and 120 h (∎), with 7 mM ABTS and 0.1 mM H_2_O_2_ in 0.1 M sodium tartrate pH 3.5, and referred to activity after 1 min incubation at pH 5. Means and 95% confidence limits are shown.

**Fig 5:**
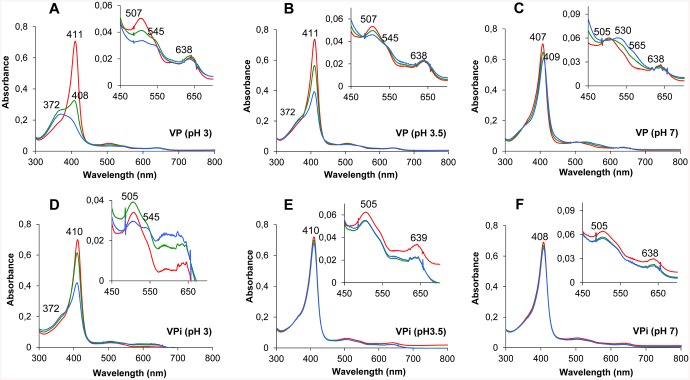
Time course of the electronic absorption spectra of native VP (top) and VPi (bottom) at acidic and neutral pH. UV-visible spectra of native VP and VPi after 0 (red line), 1 (green line) and 5 h (blue line) incubation at pH 3 (**A** and **D**), 3.5 (**B** and **E**) and 7 (**C** and **F**) in 0.1 M B&R buffer, at 25°C.

The stability of native VP and its mutated variants was evaluated during incubation at pH 3, 3.5 and 7, both by measuring the residual activity (Fig 4) and by monitoring the evolution of the UV-visible spectra (Fig 5). The decrease of the Soret band at 407 nm, typical of a stable native VP at pH 5 [14], was followed as an indicative of the integrity of the heme environment (S2 Fig). The results revealed that VPi is significantly more stable than native VP at acidic and neutral pH. The 7-fold stability improvement observed after 1 h of incubation at pH 3 was very limited in time since both the native enzyme and the mutated variant were nearly completely inactivated after 4 h of incubation (Fig 4A and 4B). By contrast, the improvement at pH 3.5 and 7 was more extended in time. VPi retained 61% (at pH 3.5) and 55% (at pH 7) of the initial activity after 24 and 120 h, respectively, compared with the native enzyme which resulted almost completely inactivated under the same experimental conditions.

This stability improvement, measured as a percentage of the initial activity, could be correlated with the increased stability of the heme environment as revealed from the analysis of the electronic absorption spectra obtained for native VP and VPi. Although the two enzymes suffered strong changes in their UV-visible spectra at pH 3 (Fig 5A and 5D), these changes were quantitatively less significant for VPi. A faster decrease in the intensity at 411 nm (Soret band) and 507 nm (CT2 band) was observed for native VP (Fig 5A), with the Soret maximum progressively blue-shifting (39 nm) to attain a maximum at 372 nm after 5 h. A shoulder at 545 nm (β band) and a maximum at 638 nm (CT1 band) were observed over the entire time of the experiment in both native VP and VPi. The spectrum described for the native VP after 5 h at pH 3 (exhibiting maxima at 372, 507, 545 and 638 nm) is compatible with that of a four-coordinate heme. During these 5 h, VPi experienced a 38% decrease in the maximum at 410 nm with a shoulder appearing near the 372 nm region (Fig 5D). This spectrum has intermediate characteristics between those of the native VP incubated at pH 3 and those previously described for the native VP at pH 4.5 (maxima at 409, 505 and 638 nm) [33] at which the enzyme has been described to be a stable high-spin heme protein. These spectral characteristics suggest that at least two VPi species are present under these conditions, one with the iron four-coordinated, and the other with the iron also coordinated by the proximal histidine. This indicates that VPi moves much more slowly than the native enzyme towards destabilization of the heme environment at pH 3.

On the other hand, only slight modifications were observed in the spectra of VPi both at pH 3.5 and 7 after 15 h of incubation (Fig 5E and 5F) unlike what happened with the native enzyme (Fig 5B and 5C). Native VP experienced a sharp drop in the Soret band at pH 3.5, following the same spectral evolution described for the enzyme incubated at pH 3, although with less dramatic changes (Fig 5B and S2B Fig). The spectral changes at pH 7 exhibited a different behavior. A slight drop and shift of the Soret band to 409 nm were observed concomitantly with both the progressive disappearance of the CT2 and CT1 bands (at 505 and 638 nm respectively), and the increasing of α and β bands at 565 and 530 nm at this pH (Fig 5C and S2C Fig) suggesting an hexacoordinated heme iron. Interestingly, the almost undetectable changes in the spectrum of VPi at pH 3.5 and 7 contrast with the loss of 39% (at pH 3.5) and 45% (at pH 7) of the initial activity after 24 h. All these results together suggest that the mutations introduced at residues exposed to the solvent in VPi ultimately contribute to increase the stability of the heme environment, necessary for activity of ligninolytic peroxidases. However, this does not seem to be enough to completely stabilize the enzyme.

VPi-br harboring seven additional basic residues exposed to the solvent, and VPi-ss containing two cysteines added to form an additional extra disulfide bond, further improved the stability of VPi at acidic pH (Fig 4C and 4D, respectively). Both variants retained 86% and 85% of the initial activity at pH 3.5 after 24 h of incubation compared to the 61% retained by VPi and far from the 1% of the native VP. Moreover, although they were not so stable at pH 3 as at pH 3.5, VPi-br and VPi-ss maintained 62% and 47% of the initial activity, respectively, at pH 3 after 1 h compared to the 38% showed by VPi and the 5% of native VP. The analysis of the UV-visble spectra of these two variants over time evidenced an evolution of the Soret band similar to that observed for VPi (S2 and S3 Figs).This is indicative of the integrity of the heme and its environment at acidic pH. Unlike what observed at low pH, VPi-ss only exhibited a slight increased stability after 1 h incubation at pH 7 compared with VPi (Fig 4D). Both VPi-br and VPi-ss not only did not show an additional improvement at longer times at neutral pH but, on the contrary, VPi-br significantly reversed the improvement attained in VPi (Fig 4C). The accumulation of all the mutations in the VPi-br-ss variant retained or slightly improved the stability of VPi-br and VPi-ss at pH 3 (43% of residual activity after 4 h) but worsen at pH 7 being even less stable than the native VP at this pH (Fig 4E).

Regarding thermal stability, VPi, VPi-br and VPi-ss exhibited T_50_ values between 58°C-60°C (S4 Fig). These values are similar to that found for the native VP (61°C) and higher than that obtained for VPi-br-ss that showed a T_50_ of 55°C (6°C lower than that of the native VP).

### Crystal Structure Analysis of the VP Variants

**Fig 6:**
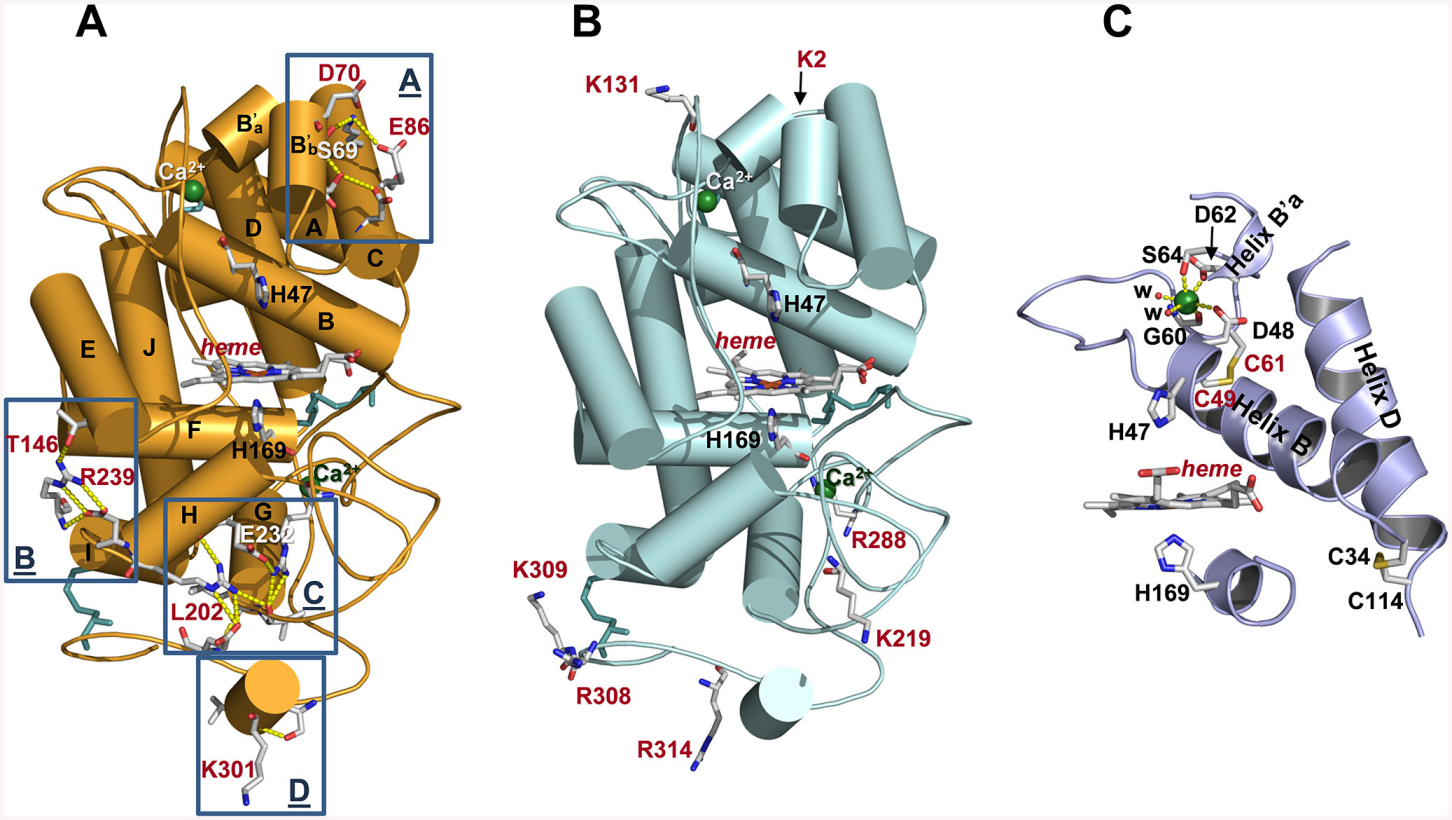
Crystal structures of VPi, VPi-br and VPi-ss variants. (**A**) Molecular structure of VPi (with 12 α-helices named from A to J, shown as cylinders) including general structural elements such as four disulfide bonds (cyan sticks) and two Ca^2+^ ions (green spheres); heme cofactor; the two catalytic histidines above and below the porphyrin plane; and mutated residues (all of them as CPK sticks) generating new H-bond and salt bridge interactions (yellow dashed lines) at four regions (named A to D) described in more detail in Fig 2. (**B**) Molecular structure of VPi-br, showing the same general elements described for VPi plus the seven solvent-exposed basic residues characterizing this variant (mutations described in VPi are also included in VPi-br but they have not been represented for simplifying purposes). (**C**) Structural detail of the VPi-ss variant showing the extra disulfide bond (formed by Cys49 and Cys61) that connects helices B and B'a (shown as cartoons); the amino acid residues (CPK sticks) and water molecules (w) coordinating the distal Ca^2+^ ion; and one of the four disulfide bonds naturally existing in native VP between cysteine residues 34 and 114 that connects helices B and D (also depicted as cartoon) (heme and axial histidines are also shown).

Crystal structures of VPi, VPi-br and VPi-ss showing enhanced pH stability were solved (Table 1) and subsequently analyzed both to verify that the mutations introduced had generated the expected stabilizing motifs and to explain how these motifs contribute to increase the enzyme stability. The overall folding characterizing the native VP, as well as the key elements that maintain its molecular architecture (i.e. two structural Ca^2+^ ions and four disulfide bonds), and position of the heme group, appear conserved in the three mutated variants (Fig 6A shows these elements in VPi). The structural changes observed are in general restricted to the regions where mutations were introduced.

VPi includes eight mutations distributed into four solvent-exposed regions (named A-D in Fig 6A). Region A (containing mutations D69S/T70D/S86E) is located at the heme distal side above the heme plane, whereas regions B, C and D (containing mutations D146T/Q239R, Q202L/H232E and S301K, respectively) are found at the proximal side below the heme plane. The three mutations introduced in region A fail to emulate the contacts identified in MnP4 (Fig 2A, left). However, compared with the native VP (Fig 2A, middle), they contribute to reinforce the interaction between helices B'_b_ and C by increasing the H-bond network in this area, as shown in the crystal structure (Fig 2A, right). Similarly, the two substitutions in region B strengthen the loop between helices H and I by interaction of the Arg239 guanidinium group with the Asp237 carboxylate (Fig 2B, right), mimicking that observed between Arg245 and Asp243 in MnP4 (Fig 2B, left). In addition, the two mutated residues in this region (Thr146 and Arg239) are able to retain the H-bond that connects the loop between helices H and I with the N-terminal end of helix E established between Asp146 and Gln239 in the native VP (Fig 2B, middle). Regarding the region C, the introduction of a glutamate at position 232 in helix H promotes the formation of a salt bridge between this amino acid and Arg227 (Fig 2C, right) emulating that observed between Glu238 and Arg233 in MnP4 (Fig 2C, left). This interaction, not existing in the native enzyme (Fig 2C, middle), reinforces an extensive H-bond network that anchors the helix H both to the C-terminal end of helix G and to Glu304 located at the C-terminal region of the protein consisting of 66 residues without clearly defined secondary structures (except for two 3-amino acids β strands and a single turn 3_10_ helix). Finally, unlike what was described for the other regions, the S301K substitution included in region D (Fig 2D, right) do not have the expected effect. This should consist in formation of a new H-bond, as observed in MnP4 (Fig 2D, left). By contrast, the side-chain of Lys301 appears exposed to the solvent. Summarizing, three of the four protein regions containing mutations exhibit a modified distribution of hydrogen bonds and salt bridges that can be correlated with the improvement in pH stability previously described for the VPi variant.

VPi-br and VPi-ss crystal structures exhibited minor differences compared with VPi regarding the interactions involving the mutated residues in region A (VPi-br also regarding the interactions of residues in region C), and no significant changes were identified in the other regions described. The analysis of the VPi-br crystal structure also revealed that the seven solvent-exposed basic residues introduced in this variant present a configuration similar to that observed for the homologous residues in MnP4. Their side-chains appear interacting with water molecules through hydrogen bonds or do not present any interaction, and no contacts are observed with surrounding residues (Fig 6B). Finally, the VPi-ss structure confirmed that the two cysteines introduced at positions 49 and 61 form an extra (fifth) disulfide bond next to the distal Ca^2+^ binding site (Fig 6C). This region did not show any additional change compared with the native enzyme even though the two new cysteines occupy positions adjacent to Asp48, Gly60, Asp62 and Ser64 involved, together with two water molecules, in Ca^2+^ coordination.

## Discussion

Over the last years several approaches have been addressed at molecular level with the aim to solve the main problems preventing the use of ligninolytic peroxidases in biotechnological applications, including high-yield production and stability to different factors [34, 35]. Some properties of the ligninolytic peroxidases have been also improved by introducing structural determinants suggested to be responsible for thermal, pH and oxidative stability [36–38], and similar results have been attained by directed molecular evolution using appropriate selection pressures [35, 39].

Recently, the development of next generation sequencing methods has allowed to sequence a large amount of genomes of ligninolytic basidiomycetes and a new array of high redox potential peroxidases has emerged [40]. Some of these new peroxidases have proved to be particularly stable under certain conditions and are being used as protein scaffolds that can be redesigned with the aim to provide enzymes with catalytic properties of interest [41]. A recent study focused on the analysis of the *P*. *ostreatus* genome sequence obtained at JGI enabled the identification and characterization of the complete set of lignin-degrading peroxidases in this white-rot fungus [8, 42], revealing strong differences in their stability properties and providing enzymes of biotechnological interest. Among these peroxidases, MnP4 has demonstrated to be especially stable at both acidic and moderately alkaline pH, and the same has been shown for a few other MnPs [7]. By contrast, VPs are usually not so stable to pH. An example is VP from *P*. *eryngii* which is easily inactivated at neutral and basic pH as well as below pH 4 [43]. VPs are more interesting than MnPs as industrial and environmental biocatalysts due to their wide substrate specificity [19, 44–48]. That is the reason why we decided to take advantage of the structural similarities between MnP4 from *P*. *ostreatus* [8] and the best characterized VP from *P*. *eryngii* [16] to identify the structural determinants responsible for such stability in MnP4 and transfer them to VP designing a more stable peroxidase of biotechnological interest.

The fact that only minimal changes are produced in the UV-visible spectrum of MnP4 incubated under acidic (pH 3) and moderately alkaline (pH 8) conditions has been reported to be indicative of the high stability of its heme environment [8]. Unlike MnP4, two different pH-induced structural transitions were identified from the analysis of the electronic absorption spectra of the native VP incubated at acidic and neutral pH at which the enzyme is inactivated. On one hand, the spectral changes at low pH suggested that the interaction between the heme iron and the imidazole group of the proximal histidine is broken. This assumption was based on the high similarities observed between the UV-visible spectrum here obtained for native VP (with maxima at 372, 507, 545 and 638 nm) and that reported for an intermediate form of metmyoglobin (maxima at 370, 510, 545 and 640) in which this cleavage is produced during the acid transformation of the native state into an unfolded form [49, 50]. Similar spectra were also obtained for horseradish and *Coprinopsis cinerea* peroxidases incubated at very low pH, and the same conclusions regarding the weakening and/or rupture of the histidine-iron bond were reached [51, 52]. On the other hand, the spectrum at neutral pH was characteristic of a VP with an hexacoordinated low-spin heme-iron [53]. According to previous studies, this form of the enzyme is the result of the formation of a bis-histidyl heme iron complex, in which both proximal and distal histidines are involved, due to loss of one or the two structural Ca^2+^ ions upon thermal [54, 55] or alkaline [56, 57] inactivation. An exhaustive characterization of a Ca^2+^-depleted VP has been reported revealing that, although it can be activated by H_2_O_2_, its redox potential and catalytic activity are dramatically affected [53].

Four variants (VPi, VPi-br, VPi-ss and VPi-br-ss) were designed to improve the pH stability of VP by introducing combinations of mutations at different molecular regions, including: i) the amino acid residues responsible for the structural determinants (extra hydrogen bonds and ion pairs) identified in MnP4 as putatively involved in its high stability towards pH; ii) basic residues surface-exposed in MnP4 that are absent in VP; and iii) two cysteines to form an additional disulfide bond not present in MnP4, nor in other ligninolytic peroxidases, but described to play a stabilizing role at high temperature and pH in an engineered MnP [36, 37]. The analysis of the crystal structures of three of these VP variants (VPi, VPi-br and VPi-ss) confirmed the presence of the mutated residues and the structural determinants engineered. Consequently, they could be definitively related with the changes observed in enzyme stability.

Major improvements in stability at acidic and neutral pH resulted from the mutations introduced in VPi (also included in VPi-br, VPi-ss and VPi-br-ss). These mutations are responsible for extra hydrogen bond and salt bridge interactions in four specific regions exposed to the solvent. The introduced residues are located in key positions, anchoring different elements of the secondary structure. At the heme distal side, the reinforced interactions between helices B'_b_ and C covering helix B and distal Ca^2+^ binding site seem to stabilize the position of the distal histidine (located at helix B) involved in enzyme activation by H_2_O_2_ [58]. Similarly, the reinforced interactions between helices E, G, H, I, and a portion of random coil, all of them covering helix F at the heme proximal side, seem to be responsible for the stabilization of the proximal histidine (located at helix F) acting as the fifth heme iron ligand. The stabilization of the environment of this residue is critical taking into account that the strength of the interaction between this histidine and the heme iron has been proposed as one of the factors determining the high redox potential of ligninolytic peroxidases [59, 60]. In short, this analysis shows how mutations reinforcing specific regions of the overall structure ultimately contribute to stabilize the architecture of the heme pocket located inside the protein. Stabilization of this pocket is crucial since the redox potential and activation of peroxidases by H_2_O_2_ depend on the precise position of the above two histidines located immediately below and above the heme cofactor. This stabilization was definitively confirmed by the spectral analysis of VPi showing a stable pentacoordinate high-spin heme-iron state at pH 3.5 and 7 characteristic of an active peroxidase [14], unlike what observed for the native enzyme, where the breakdown of the proximal histidine-iron interaction (at pH 3–3.5) and iron hexacoordination by proximal and distal histidines (at pH 7) was produced.

In spite of the stabilization of the heme pocket, partial loss of activity was observed for VPi at pH 3.5 and pH 7 over time. Therefore, this is not enough to completely stabilize the enzyme, and structural changes affecting other protein regions are most probably produced both at acidic and neutral pH. The structural changes observed in MnP4 when incubated at pH 8 [8] support this idea. These changes were related with the loss of ~ 15% activity even though its UV-visible spectrum, and in consequence the heme environment, were completely stable.

A stable heme pocket was also observed in VPi-br, VPi-ss and VPi-br-ss at pH 3 and 3.5 as inferred from the analysis of the spectra and time course of their Soret maximum. These three variants contain those mutations previously described to stabilize the heme environment in VPi plus additional substitutions responsible for further stability improvements at acidic pH (basic residues in VPi-br, an extra disulfide bond in VPi-ss and both basic residues and a disulfide bond in Vpi-br-ss). We decided to design the VPi-br variant because the high number of basic residues exposed to the solvent identified in MnP4 led us to think that they could be also responsible for the stability of this enzyme at low pH. No other ligninolytic peroxidases from *P*. *ostreatus*, all of them less stable than MnP4 [8], nor VP from *P*. *eryngii* (including a total of 9 lysines and 9 arginines), have a similar number of basic residues with their ionizable side chains oriented to the solvent. The introduction of basic residues, mainly arginines, at the molecular surface has been described to improve pH stability [61, 62] as well as thermostability and other enzyme properties, including optimal temperature and pH, catalytic efficiency [63, 64], and stability to chemical denaturants [62]. In our case, the increased stability of VPi-br at acidic pH compared with VPi could be explained by a general stabilizing effect of the extra basic residues participating in charge-charge interactions with other charged amino acids of the protein surface (although these interactions were not observed in the VPi-br crystal structure) and by interacting with the solvent improving the solubility of the protein. It should be also noted that the polar surface of the VPi-br variant is significantly increased with respect to VPi by replacing five hydrophobic residues (four alanines and leucine) with polar charged amino acids (four arginines and one lysine).

Regarding VPi-ss, the extra disulfide bond included in this variant further stabilized the enzyme at pH 3.5 by reinforcing the molecular structure at the distal heme side together with mutations D69S/T70D/S86E, whose stabilizing role in VPi has been described above. Cysteines forming the new disulfide bond (Cys49-Cys61) are located in a critical position. They are next to the residues involved in coordination of the distal Ca^2+^ ion (Asp48, Gly60, Asp62 and Ser64), which are adjacent to the position of the distal histidine (His47) located at helix B. This disulfide bond stabilizes a long 14 amino acids loop connecting helices B and B'a, and gives rigidity to the helix B, which remains anchored by two disulfide bonds (the new one at its N-terminal end, and that formed by Cys34-Cys114 binding the C-terminal ends of helices B and D) (Fig 6C). In short, the extra disulfide bond contributes to stabilize the position of the distal histidine in helix B. This analysis, together with that previously performed for VPi, could explain the accumulative effect due to this disulfide bond and the extra hydrogen bond and salt bridge interactions on the VPi-ss stability improvement observed at pH 3.5. We expected a similar effect at pH 7 according to the results reported for an engineered MnP of *Phanerochaete chrysosporium* including a disulfide bond at the same position [36]. At this pH, the structural destabilization produced by the release of the distal Ca^2+^ ion should be compensated by the presence of the extra disulfide bond. However, VPi-ss did not show increased stability at neutral pH compared with VPi, unlike what was observed at pH 3.5. These differences again confirm that different mechanisms are eventually responsible for pH inactivation at acidic and neutral pH.

Thermal inactivation of ligninolytic peroxidases has also been correlated with the release of the structural Ca^2+^ ions [54, 65]. Increased thermostability has been observed in the aforementioned engineered MnP containing an extra disulfide bond [36], and electrostatic interactions on the protein surface have also been reported to be involved in thermal stabilization [66]. However, none of the designed VP variants including these structural determinants (VPi, VPi-ss and VPi-br-ss) exhibited a T_50_ value higher than that of the native enzyme (61.2°C). This value is already high compared with those obtained for other recombinant (expressed in *E*. *coli*) ligninolytic peroxidases (T_50_ ranging from 56.8 to 38°C after 10 min incubation) [7, 8, 67], and even compared with glycosylated wild-type LiP and MnP from *P*. *chrysosporium* [67]. Moreover, thermal stability of native VP (88% residual activity after 30 min incubation at 37°C, this work) was similar to that of LiP variants containing ancestral residues, which were recently designed with the aim of improving the thermal stability of this enzyme [68]. This means that VP used in this work is naturally thermostable compared with other ligninolytic peroxidases. However, directed molecular evolution experiments have resulted in a VP variant with a T_50_ improvement of 8°C over the parental type [35], showing that there is still some room to improve the VP thermal stability by protein engineering.

Something interesting from an applied perspective is the effect observed on the catalytic properties due to the mutations introduced. Affect them as little as possible was a premise of this work, and that was the reason why all substitutions were introduced far from the three catalytic sites present in VP. A small negative impact difficult to rationalize with the data in hand, was observed in some cases. The most noteworthy was the shifting of the optimum pH to a more acidic value for oxidation of high redox potential substrates at the solvent exposed catalytic tryptophan [14] (VA oxidation by the four VP variants, and RB5 oxidation by VPi and VPi-ss). Two variants (VPi and VPi-ss) also improved its ability to oxidize low redox potential substrates (ABTS) at the main heme access channel [15] at a lower pH compared with the native enzyme at its optimum pH. A similar shifting has been reported for a long MnP intrinsically stable at acidic pH transformed into a VP by engineering an exposed catalytic site [41]. The improvement in affinity for RB5 and ABTS at the new optima pHs suggests a better positioning of these two large sulfonated substrates at the corresponding active sites most probably due to interactions with the distant residues introduced in these variants. On the other hand, the redox potential of heme peroxidases is strongly influenced by pH [69], and different studies have shown that the oxidative activity of these enzymes increases at acidic pH [70, 71]. The fact that the designed variants are more stable at low pH make them of special interest from a biotechnological point of view in processes (e.g. ligninolysis) favored by acidic pH (due to the increased redox potential of the heme cofactor when the pH decreases).

## Conclusions

*P*. *eryngii* VP and *P*. *ostreatus* MnP4 share the same protein scaffold. The identification and subsequent transfer into VP of the structural determinants putatively responsible for the high stability towards pH of MnP4 allowed us to obtain four variants with an improved pH stability. The analysis of the crystal structures of three of them confirmed that the observed stability improvement is due to the introduction of such determinants, indirectly proving that they should also contribute to the pH stability of MnP4. A significant increased stability at both acidic and neutral pH was achieved by mutations contributing to generate extra hydrogen bond and salt bridge interactions exposed to the solvent. The stabilization of the heme pocket resulting from these interactions was enhanced at low pH by the inclusion of an extra disulfide bond. Further stabilization was also attained at acidic pH by introducing solvent exposed basic residues, probably increasing the protein solubility. In spite of the high number of mutations introduced (seventeen in VPi-br-ss), the VP variants retained the promiscuity of the native enzyme and the catalytic activity was only minimally compromised. The pH stability improvement obtained in this work, together with the intrinsic thermal stability of VP, and the reported possibility to further improve the thermal and oxidative stability of VP by protein engineering [35, 38], make this enzyme a promising biocatalyst to oxidize lignin and other high redox potential aromatic compounds. Currently, a large amount of genomes of ligninolytic basidiomycetes are being sequenced. The characterization of peroxidases encoded by these genomes will provide us with novel enzymes, like MnP4, with new catalytic properties and improved stabilities. The use of these enzymes in comparative structural analyses as that described in this work will allow to design new tailor-made biocatalysts of interest for different applications, including those aimed to the development of the Biorefinery concept that seeks the integral use of plant biomass.

## Supporting Information

S1 Fig Electronic absorption spectra of native VP and variants VPi, VPi-br, VPi-ss and VPi-br-ss. The spectra were obtained in 10 mM sodium tartrate, pH 5, at 25°C (details of the 450 nm-700 nm region are shown in x4 scale).
(TIF)

S2 Fig pH stability of native VP and its mutated variants monitored as the absorbance of the Soret band at 407 nm. VP (—), VPi (─ ─), VPi-br (─ · ─), VPi-ss (----) and VPi-br-ss (····) were incubated in 0.1mM B&R buffer at pH 3 (**A**), pH 3.5 (**B**) and pH 7 (**C**) and 25°C.
(TIF)

S3 Fig Time course of the electronic absorption spectra of VPi-br (top) and VPi-ss (bottom) at acidic pH. UV-visible spectra of Vpi-br and Vpi-ss after 0 (red line), 1 (green line) and 5 h (blue line) of incubation at pH 3 (**A** and **C**) and 3.5 (**B** and **D**) in 0.1 M B&R buffer at 25°C.
(TIF)

S4 Fig T_50_ profiles of native VP and four designed mutated variants. Residual activity was estimated from ABTS oxidation in 0.1 M sodium tartrate, pH 3.5.
(TIF)
