# Correction: Improving the pH-stability of Versatile Peroxidase by Comparative Structural Analysis with a Naturally-Stable Manganese Peroxidase

**DOI:** 10.1371/journal.pone.0143267

**Published:** 2015-11-12

**Authors:** The PLOS ONE Staff

There are errors in the Funding section. The complete, correct funding information is as follows:

This work was funded by the Commission of the European Communities through the PEROXICATS (KBBE-2010-4-265397, Novel and more robust fungal peroxidases as industrial biocatalysts) and INDOX (KBBE-2013-7-613549, "Optimized oxidoreductases for medium and large scale industrial biotransformations") projects to ATM, by the Spanish Ministerio de Economía y Competitividad (MINECO) through the HIPOP (BIO2011-26694, “Screening and engineering of new high-redox potential peroxidases”) to FJR-D, NOESIS (BIO-2014-56388-R, "New oxidative enzymes for sustainable industries") to FJR-D, BFU2011-24615 and CSD2009-00088 projects, and by the EC 7th Framework Programme (FP7/2007-2013) under BioStructx-5959 (grant agreement N° 283570). VS-J and FJR-D thank the financial support of a research fellowship (Formación de Personal Investigador, FPI) and a Ramón y Cajal contract of the Spanish MINECO, respectively. The funders had no role in study design, data collection and analysis, decision to publish, or preparation of the manuscript.

The publisher apologizes for the errors.
