# Supplementary figures and images for: Improving the pH-stability of Versatile Peroxidase by Comparative Structural Analysis with a Naturally-Stable Manganese Peroxidase
Source: PLoS One. 2015 Oct 23;10(10):e0140984. doi: 10.1371/journal.pone.0140984 (PMC4619715; doi:10.1371/journal.pone.0140984)

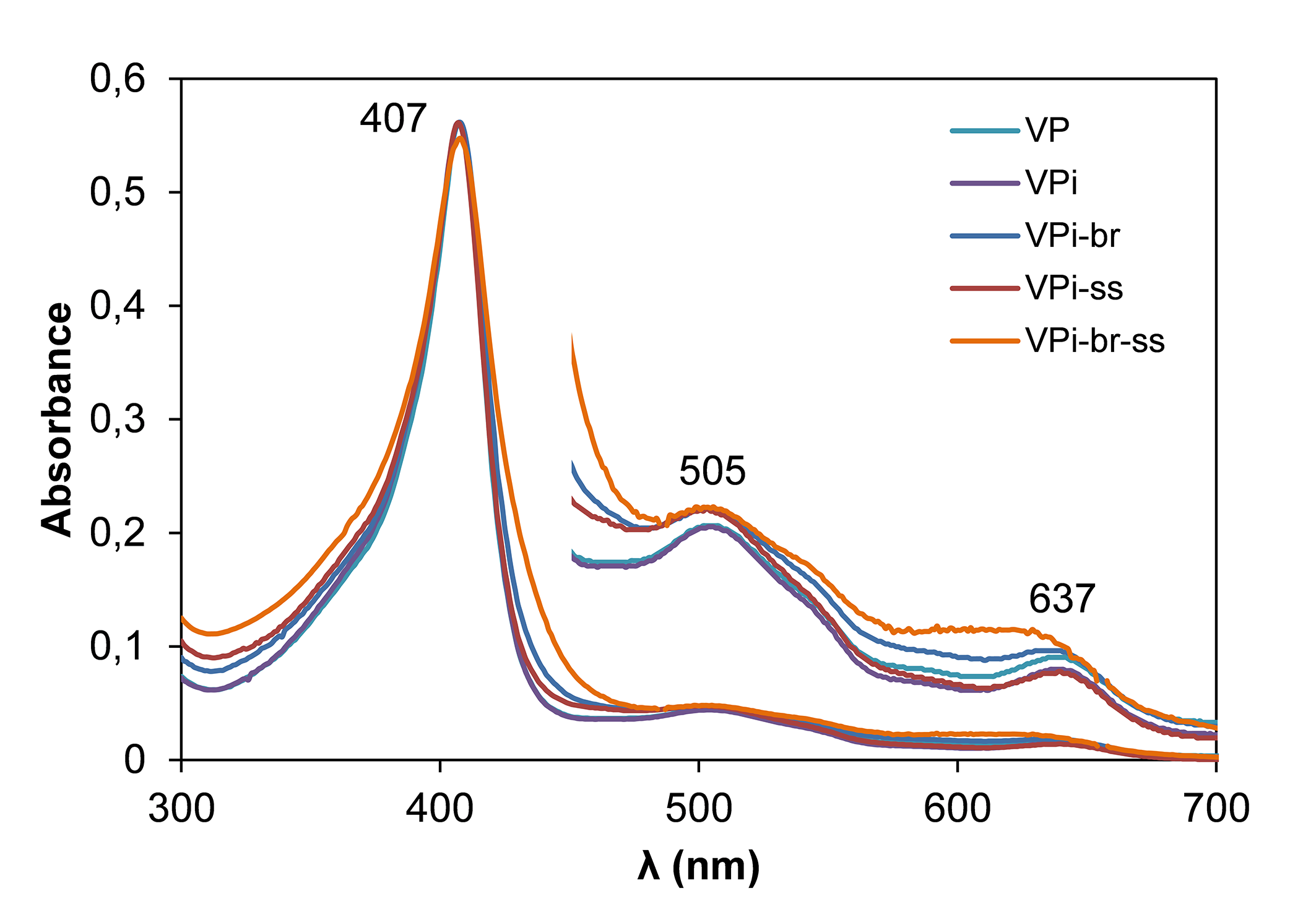

Supplement: S1 Fig — The spectra were obtained in 10 mM sodium tartrate, pH 5, at 25°C (details of the 450 nm-700 nm region are shown in x4 scale). (TIF) [file pone.0140984.s001.tif]

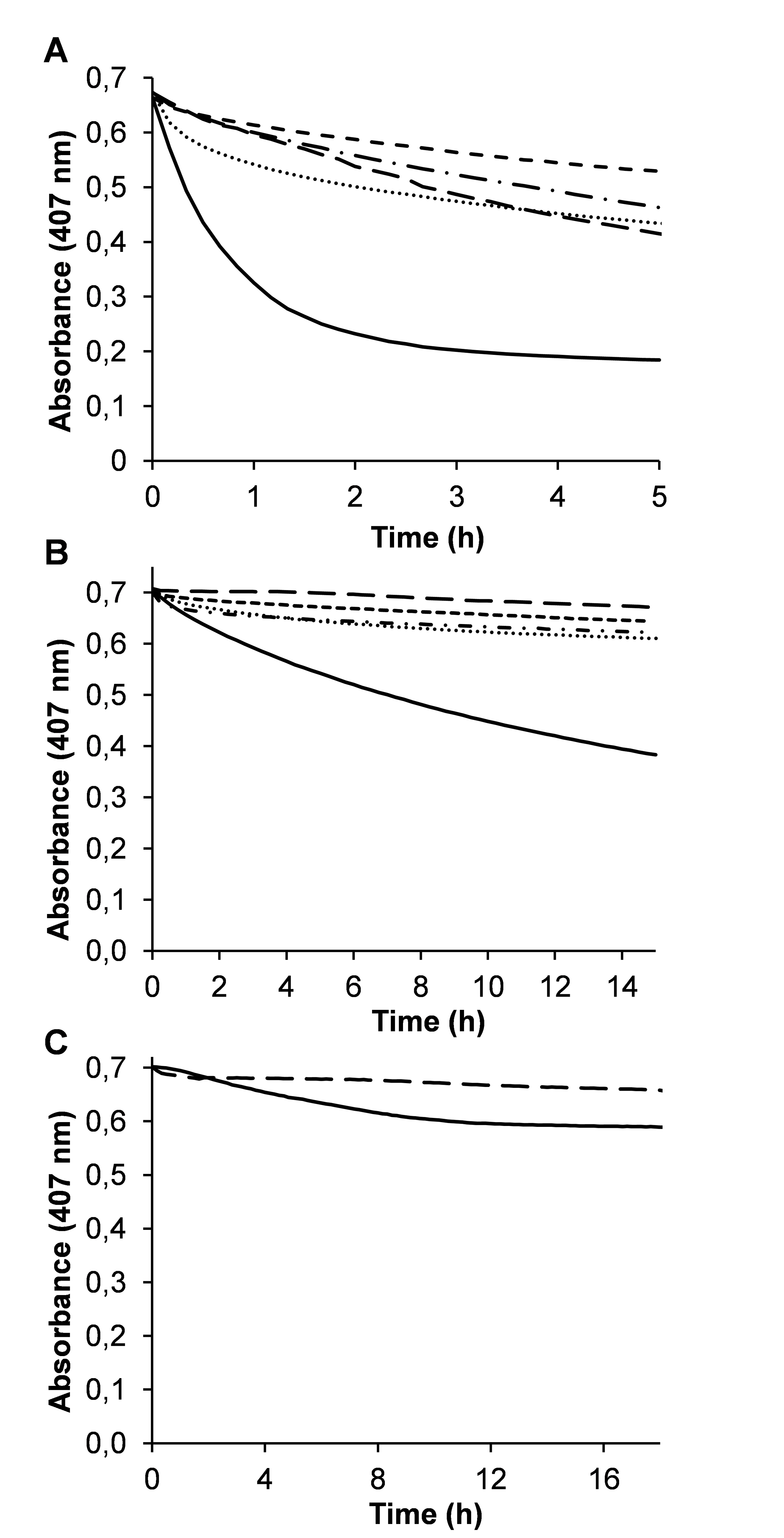

Supplement: S2 Fig — VP (—), VPi (─ ─), VPi-br (─ · ─), VPi-ss (----) and VPi-br-ss (····) were incubated in 0.1mM B&R buffer at pH 3 (A), pH 3.5 (B) and pH 7 (C) and 25°C. (TIF) [file pone.0140984.s002.tif]

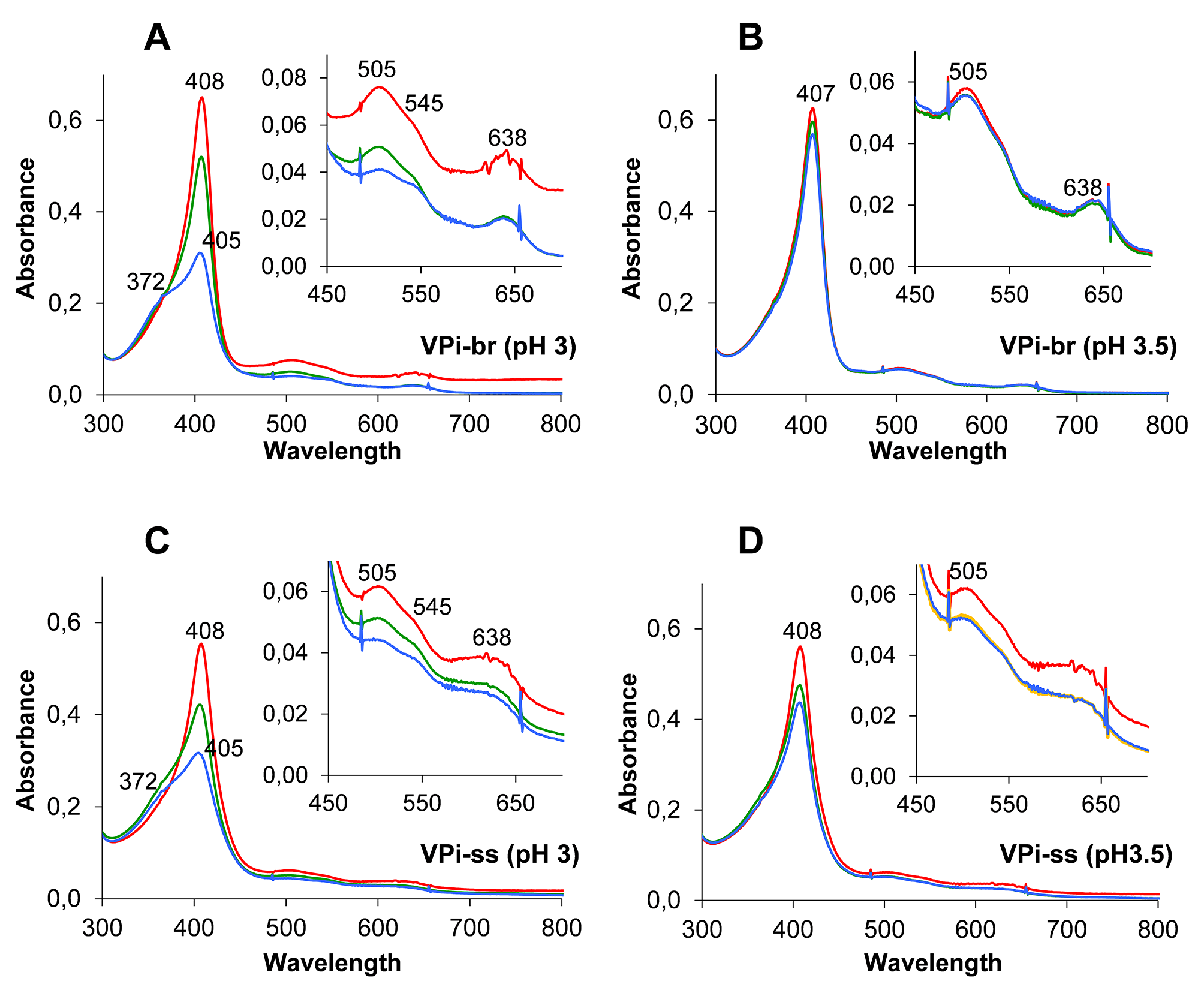

Supplement: S3 Fig — UV-visible spectra of Vpi-br and Vpi-ss after 0 (red line), 1 (green line) and 5 h (blue line) of incubation at pH 3 (A and C) and 3.5 (B and D) in 0.1 M B&R buffer at 25°C. (TIF) [file pone.0140984.s003.tif]

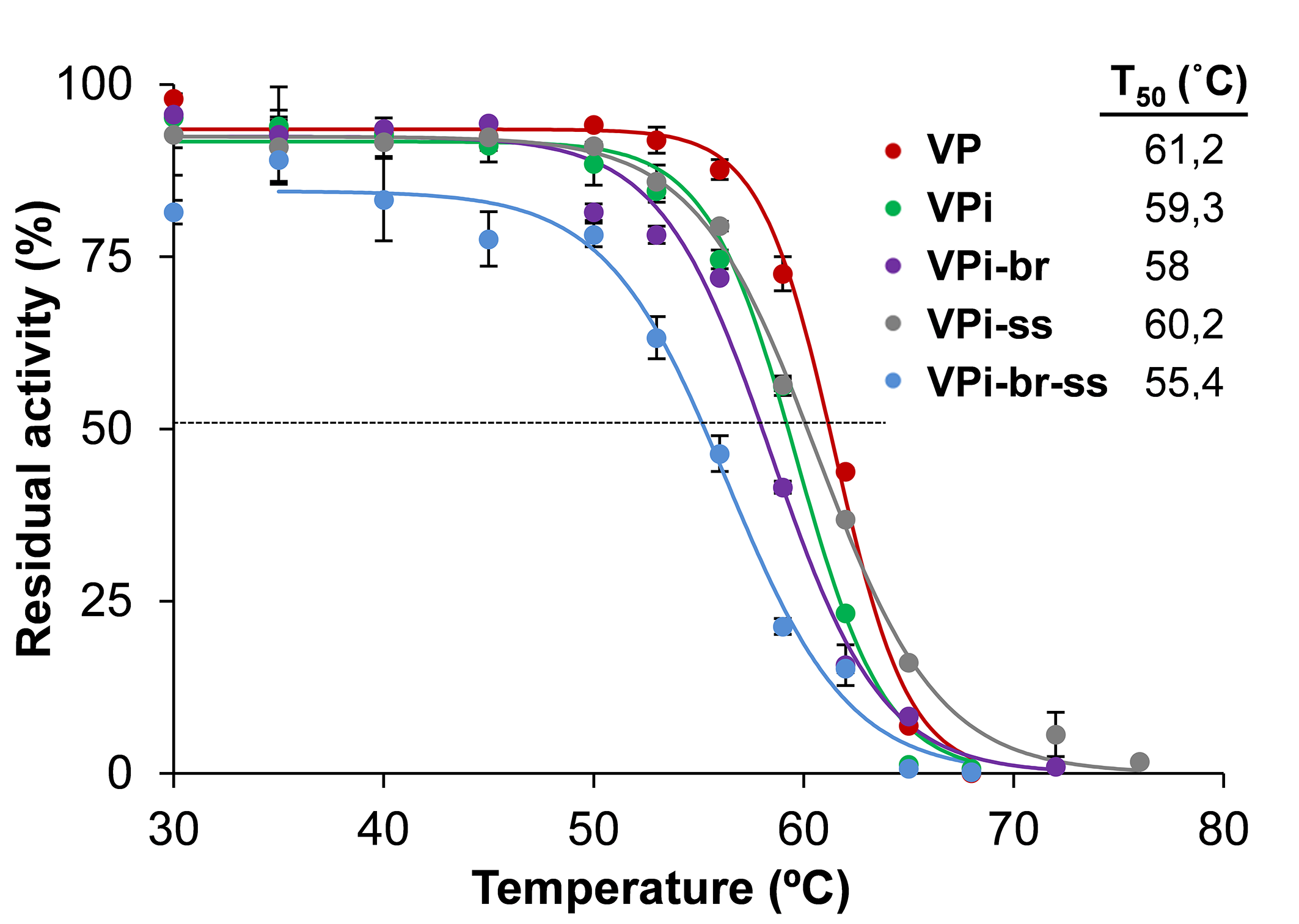

Supplement: S4 Fig — Residual activity was estimated from ABTS oxidation in 0.1 M sodium tartrate, pH 3.5. (TIF) [file pone.0140984.s004.tif]
